# Supplementary figures and images for: Bee diversity in secondary forests and coffee plantations in a transition between foothills and highlands in the Guatemalan Pacific Coast
Source: PeerJ. 2020 Jun 4;8:e9257. doi: 10.7717/peerj.9257 (PMC7276151; doi:10.7717/peerj.9257)

a

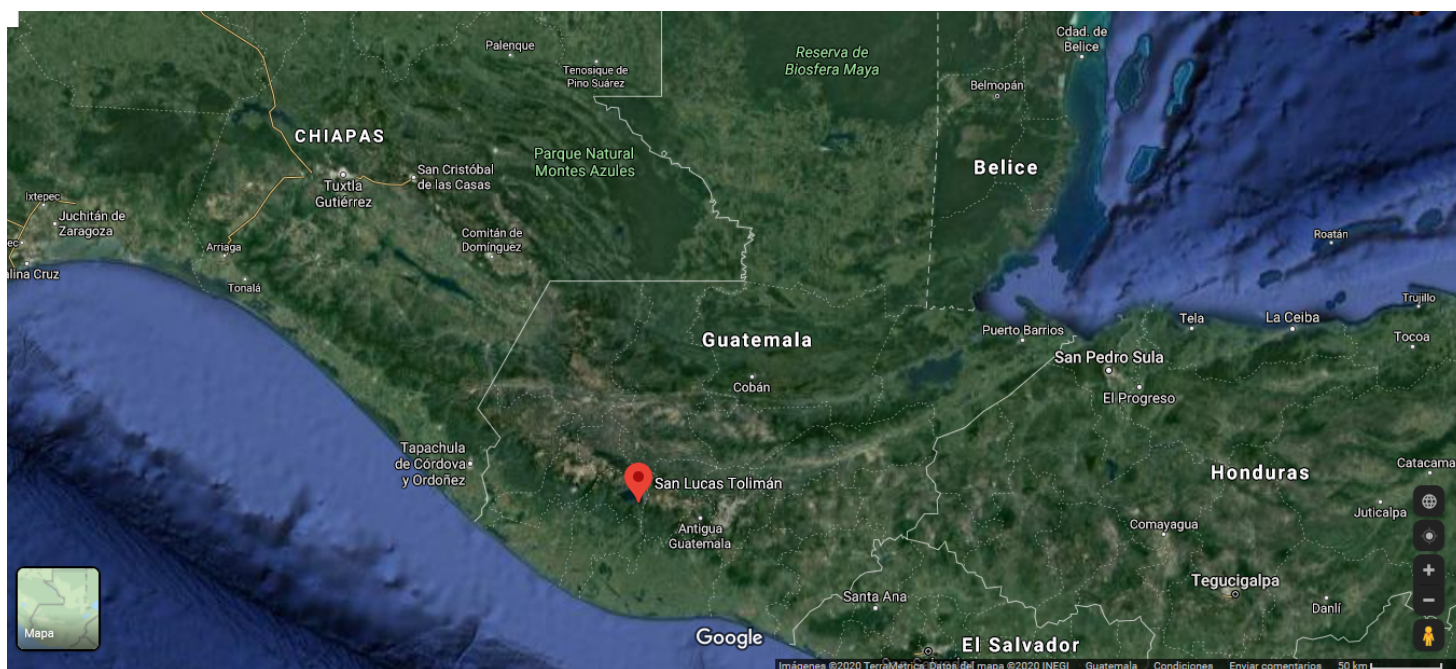

b

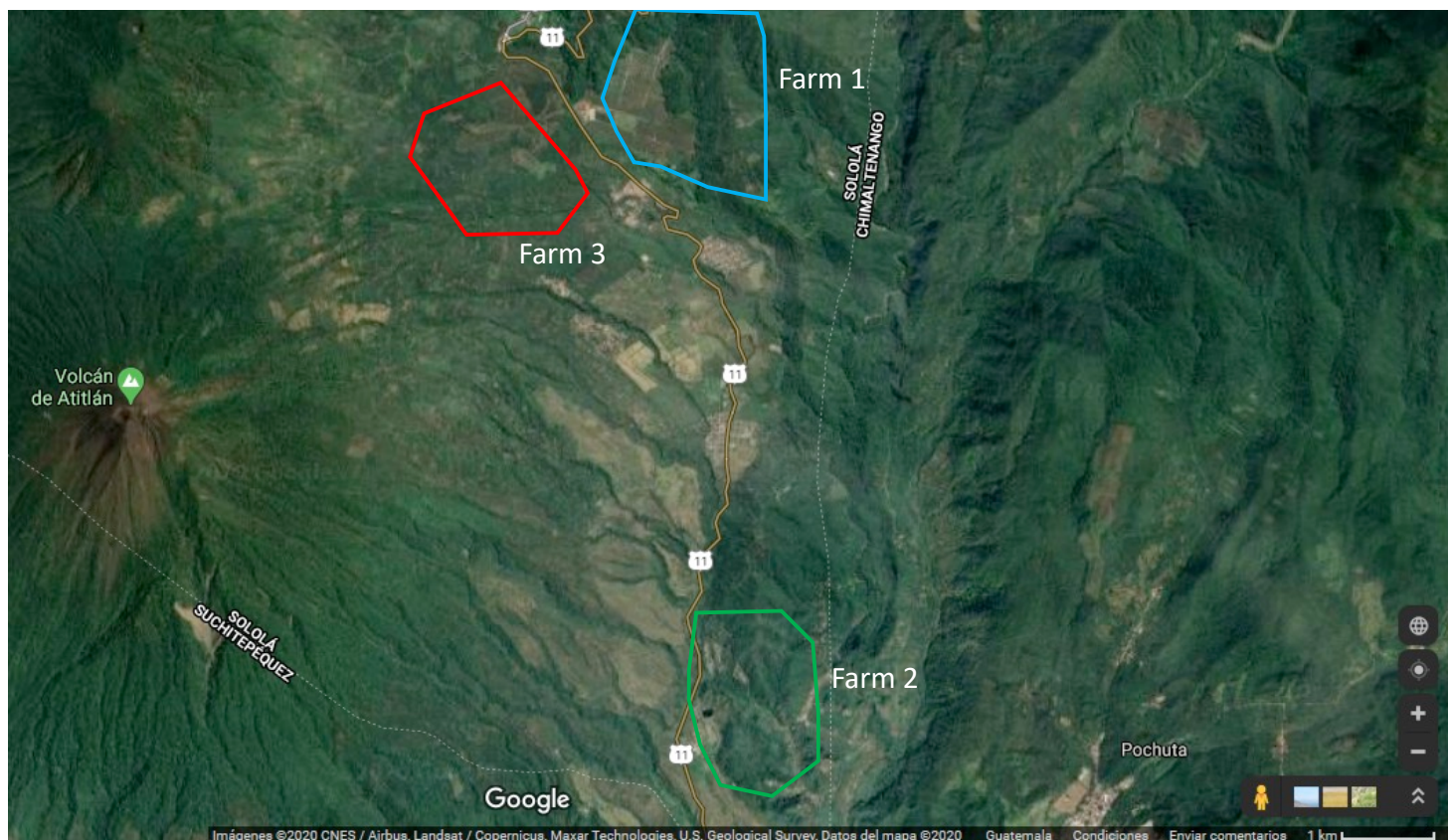

Supplement: Supplemental Information 1 — (A) Location of San Lucas Tolimán, Sololá in a Guatemala map. (B) Location map of the three studied farms. Farm 1, in the blue polygon shows a systematized plantation with a private reserve around it. Farm 2, in the red polygon, shows a not so systematized distribution closer to the volcano slopes. Farm 3, in the green polygon shows more vegetation with no coffee quadrants in sight. Map credit: Google, Imagenes (C) 2020 Terrametrics, Datos del mapa (C) 2020 INEGI; Google, Imagnes (C) 2020 CNES/Airbus, Dansat/Copernicus, Maxar Technologies US Geographical Survey, Datos del mapa (C) 2020. (Google Maps, San Lucas Tolimán, Guatemala). Retrieved 26 April 2020, from https://www.google.com.gt/maps/place/San+Lucas+Tolimán/@14.6382008,-91.1447444,4322m/data=!3m1!1e3!m5!3m4!1s0x858935e7a63274d3:0x91aff322ca7ecfe2!8m2!3d14.6355926!4d-91.1424989!5m1!1e4 [file peerj-08-9257-s001.pdf]
