# Supplementary material for: Bee diversity in secondary forests and coffee plantations in a transition between foothills and highlands in the Guatemalan Pacific Coast
Source: PeerJ. 2020 Jun 4;8:e9257. doi: 10.7717/peerj.9257 (PMC7276151; doi:10.7717/peerj.9257)
Supplement: Supplemental Information 2 [file peerj-08-9257-s002.docx]

**Appendix 2.** Bee species occurrence per plot and month.

| BEE SPECIES | FARMS | | | | | | | | | COLLECTION DATE | | | | | | | | | | | | |
| --- | --- | --- | --- | --- | --- | --- | --- | --- | --- | --- | --- | --- | --- | --- | --- | --- | --- | --- | --- | --- | --- | --- |
|  | FARM 1 | | | FARM 2 | | | FARM 3 | | | 2013 | | | | | | | | | | 2014 | | |
|  | Coffee | early | late | Coffee | early | late | Coffee | early | late | 3 | 4 | 5 | 6 | 7 | 8 | 9 | 10 | 11 | 12 | 1 | 2 | 3 |
| Agapostemon melanurus Cockerell, 1949 |  |  |  |  | 1 |  |  |  |  |  |  |  | 1 |  |  |  |  |  |  |  |  |  |
| Agapostemon texanus Cresson, 1872 |  |  |  | 1 | 6 |  |  |  |  |  |  |  |  |  |  |  |  |  |  |  | 3 | 4 |
| Andrena uyacensis Cockerell, 1949 |  |  | 1 |  |  | 2 |  |  |  |  |  |  |  |  |  |  |  | 1 | 1 | 1 |  |  |
| Andrena vidalesi Cockerell, 1949 |  |  |  |  | 3 |  |  |  |  |  |  |  |  |  |  |  |  | 3 |  |  |  |  |
| Apis mellifera Linnaeus, 1758 | 45 | 239 | 47 | 89 | 199 | 72 | 309 | 239 | 83 | 137 | 25 | 43 | 53 | 35 | 78 | 71 | 48 | 109 | 164 | 173 | 206 | 180 |
| Augochlora sidaefolia Cockerell, 1913 |  | 2 |  |  | 1 |  |  |  |  | 1 |  |  |  | 1 |  |  |  |  |  |  | 1 |  |
| Augochlora smaragdina Friese, 1917 | 1 | 1 | 1 |  | 4 |  | 2 | 2 | 1 | 2 |  |  |  |  |  |  |  | 1 | 2 | 3 | 2 | 2 |
| Augochlora sp. 1 |  |  |  |  | 1 |  |  |  |  |  |  |  |  | 1 |  |  |  |  |  |  |  |  |
| Augochlora sp. 2 |  | 1 |  |  | 17 |  | 4 |  |  |  |  | 4 |  | 2 | 1 | 1 | 1 |  | 2 | 1 | 9 | 1 |
| Augochlora sp. 3 |  |  |  |  | 2 |  |  |  |  |  |  |  |  |  |  |  |  |  |  |  | 1 | 1 |
| Augochlora sp. 4 |  |  |  |  | 5 |  | 2 |  |  |  |  |  |  |  |  |  |  |  | 1 | 1 | 2 | 3 |
| Augochlora sp. 5 |  | 1 |  |  | 1 |  | 1 |  |  |  |  | 1 |  | 1 |  |  |  |  |  |  | 1 |  |
| Augochlora sp. 6 |  | 2 |  |  |  |  |  |  |  |  |  |  | 2 |  |  |  |  |  |  |  |  |  |
| Augochlorella sp. 1 |  |  |  |  | 1 |  |  |  |  |  |  |  |  |  |  |  |  |  |  |  | 1 |  |
| Bombus mexicanus Cresson, 1878 |  |  |  |  |  |  |  | 2 |  | 1 |  |  |  |  |  |  | 1 |  |  |  |  |  |
| Bombus wilmattae Cockerell, 1912 |  |  |  |  | 5 | 1 | 1 | 3 | 3 | 1 | 2 | 1 |  |  |  | 1 | 1 | 5 | 1 |  |  | 1 |
| Caenaugochlora tonsilis Vachal, 1904 | 1 | 3 |  |  |  |  |  |  |  |  |  |  | 1 |  | 3 |  |  |  |  |  |  |  |
| Caenaugochlora (Pseudaugochlora) gramínea Fabricius, 1804 | 1 |  |  |  | 2 |  |  |  |  |  |  |  | 1 |  |  |  |  |  |  |  |  | 2 |
| Caenaugochlora sp. 1 |  |  |  |  |  | 1 |  |  |  |  |  | 1 |  |  |  |  |  |  |  |  |  |  |
| Caenohalictus sp. 1 |  |  |  |  | 3 |  |  |  |  |  |  |  | 2 |  |  |  | 1 |  |  |  |  |  |
| Centris mexicana Smith, 1854 |  |  |  |  | 1 |  |  | 1 | 1 |  |  |  |  |  |  |  |  |  |  |  | 1 | 2 |
| Centris nítida Smith, 1874 |  |  | 2 |  |  |  |  |  |  | 2 |  |  |  |  |  |  |  |  |  |  |  |  |
| Ceratina sp. 1 | 14 | 22 | 3 |  | 14 |  | 9 |  | 2 | 3 | 8 | 9 | 4 | 4 | 4 | 10 |  | 7 | 2 | 3 | 7 | 3 |
| Ceratina sp. 2 | 1 | 4 |  |  | 17 |  | 1 | 2 |  |  |  |  |  | 1 | 1 |  | 1 | 3 | 8 | 4 | 2 | 5 |
| Ceratina sp. 3 |  |  |  |  | 1 |  |  |  |  |  |  |  |  | 1 |  |  |  |  |  |  |  |  |
| Ceratina sp. 4 |  |  |  |  | 4 |  |  |  |  |  |  |  |  |  | 1 |  |  |  |  | 1 | 2 |  |
| Ceratina sp. 6 | 8 | 7 |  |  | 94 |  | 4 |  |  |  | 5 | 7 | 2 | 41 | 1 | 3 |  | 26 | 3 | 12 | 2 | 11 |
| Ceratina sp. 7 | 3 | 4 | 2 |  | 25 |  | 3 |  |  | 1 | 2 | 6 | 3 | 5 | 1 |  |  | 5 | 2 | 3 | 1 | 8 |
| Chilicola ashmeadi Crawford, 1906 |  |  |  |  |  | 2 |  |  |  | 2 |  |  |  |  |  |  |  |  |  |  |  |  |
| Coelioxys azteca Cresson, 187 | 2 | 1 |  |  | 1 |  |  |  |  |  |  | 1 | 2 |  |  |  |  |  |  |  | 1 |  |
| Coelioxys sp. 1 | 1 |  |  |  | 1 |  |  |  |  |  |  |  |  |  | 1 |  |  |  |  |  | 1 |  |
| Colletes sp.A |  |  | 1 |  | 7 |  |  |  |  |  |  |  |  |  |  |  | 1 |  |  |  | 3 | 4 |
| Colletes sp.B |  |  |  |  |  |  |  |  | 1 |  |  | 1 |  |  |  |  |  |  |  |  |  |  |
| Colletes sp.C |  |  |  |  | 1 |  |  |  |  |  |  |  |  |  |  |  |  |  |  |  |  | 1 |
| Euglossa atroventa Dressler, 1978 |  | 1 |  |  |  |  |  |  |  |  |  |  |  | 1 |  |  |  |  |  |  |  |  |
| Euglossa imperialis Cockerell 1923 | 1 |  | 1 |  |  |  |  |  |  |  |  |  |  |  | 1 |  | 1 |  |  |  |  |  |
| Euglossa obtusa Dressler, 1978 |  | 1 |  |  |  |  |  |  |  |  | 1 |  |  |  |  |  |  |  |  |  |  |  |
| Euglossa variabilis Friese, 1899 |  | 4 |  |  |  |  |  |  |  | 2 | 2 |  |  |  |  |  |  |  |  |  |  |  |
| Euglossa viridissima Friese, 1899 |  | 1 |  |  |  |  |  | 1 |  |  | 1 | 1 |  |  |  |  |  |  |  |  |  |  |
| Eulaema polycroma Mocsáry, 1899 |  |  |  |  |  |  |  | 2 |  |  |  | 1 |  |  |  |  |  |  |  |  |  | 1 |
| Exomalopsis sp. 1 |  |  |  |  | 8 | 1 |  |  |  |  |  |  | 2 |  | 3 | 1 |  |  | 1 | 1 |  | 1 |
| Halictus hesperus Smith, 1862 |  | 1 | 1 |  |  |  |  |  |  |  |  |  |  |  |  |  |  |  |  |  | 2 |  |
| Heriades (Neotrypetes) rufapicata Cockerell, 1949 |  |  | 1 |  | 1 |  |  |  |  |  |  |  |  |  |  |  |  |  |  |  | 1 | 1 |
| Pseudopanurgus crenulatus Cockerell, 1905 | 4 |  |  |  | 30 |  | 1 |  |  |  |  |  |  |  | 11 | 7 | 5 | 5 | 1 |  | 3 | 3 |
| Heterosarus sp. 1 |  |  |  | 3 | 18 |  | 15 |  |  |  |  |  |  | 1 |  | 2 | 9 | 21 | 3 |  |  |  |
| Hylaeus sp. 1 | 9 | 4 | 3 |  | 15 |  | 2 |  |  |  | 2 | 3 | 8 | 2 |  | 9 |  | 2 |  |  | 2 | 5 |
| Lasioglossum (Dialictus) aurora (Smith); Moure & Hurd 1987: 91 |  |  |  | 2 | 1 |  |  |  |  |  |  |  |  |  | 1 |  | 2 |  |  |  |  |  |
| Lasioglossum (Dialictus) sp. 1 |  | 1 |  |  | 5 |  |  |  |  |  |  | 1 | 1 | 2 | 1 |  |  |  |  |  |  | 1 |
| Lasioglossum (Dialictus) sp. 2 |  | 12 | 3 | 1 | 24 | 1 | 18 |  | 1 | 2 | 4 | 11 |  | 2 | 3 | 3 | 4 | 5 | 12 | 7 | 3 | 4 |
| Lasioglossum (Dialictus) sp. 3 |  |  |  |  | 1 |  |  |  |  |  |  |  |  |  |  |  |  |  |  |  |  | 1 |
| Lasioglossum (Dialictus) sp. 4 |  | 3 | 1 |  | 3 |  |  |  | 3 |  |  | 1 | 3 | 1 | 2 |  |  | 2 |  | 1 |  |  |
| Lasioglossum (Dialictus) sp. 5 |  |  |  |  | 2 |  |  |  |  |  |  |  |  |  |  |  |  |  |  |  |  | 2 |
| Lasioglossum (Dialictus) sp. 6 |  | 1 |  |  | 3 |  |  | 1 | 1 |  |  | 1 |  | 1 |  |  |  | 2 | 1 |  | 1 |  |
| Lasioglossum (Evylaeus) sp. 1 |  | 4 |  | 1 | 10 |  | 1 |  |  | 3 |  | 1 |  |  |  | 1 |  |  | 3 | 4 | 1 | 3 |
| Lasioglossum (Evylaeus) sp. 2 |  |  |  |  | 2 |  |  |  |  |  |  |  |  |  |  |  |  |  |  |  | 1 | 1 |
| Lasioglossum (Evylaeus) sp. 3 |  | 1 |  |  | 14 |  | 1 |  | 1 |  |  | 4 | 3 | 1 | 2 |  | 1 | 2 | 2 |  | 2 |  |
| Lasioglossum (Lasioglossum) crocoturum Vachal, 1904 |  |  |  |  | 6 |  |  |  |  |  |  |  |  |  |  |  |  |  |  |  | 2 | 4 |
| Lasioglossum (Lasioglossum) sp. 1 |  |  |  |  | 1 |  |  |  |  |  |  |  |  |  |  |  | 1 |  |  |  |  |  |
| Lasioglossum (Lasioglossum) sp. 2 |  |  |  |  |  | 1 |  |  |  |  |  | 1 |  |  |  |  |  |  |  |  |  |  |
| Lasioglossum (Dialictus) verapaz Engel, Hinojosa-Díaz & Yáñez-  Ordóñez, 2007 |  |  |  |  | 1 |  |  |  |  |  |  |  |  | 1 |  |  |  |  |  |  |  |  |
| Megachile sp. 1 | 2 | 6 | 1 |  | 6 | 3 |  |  | 3 |  | 1 | 3 | 1 |  | 3 |  | 3 | 2 | 4 |  | 3 | 1 |
| Megachile sp. 2 |  | 1 |  |  | 1 |  |  |  | 1 |  |  |  |  |  |  |  | 1 | 1 |  |  | 1 |  |
| Megachile sp. 3 |  | 6 |  |  | 1 |  |  | 7 |  |  |  | 4 | 2 |  |  |  | 1 | 3 | 2 |  | 1 | 1 |
| Megachile sp. 5 | 1 | 2 | 1 |  | 4 |  |  | 2 |  |  |  | 1 | 1 |  |  |  | 2 |  |  |  | 5 | 1 |
| Megachile sp. 6 |  |  |  |  | 1 |  |  |  |  |  |  |  |  |  |  | 1 |  |  |  |  |  |  |
| Megachile sp. 7 |  | 2 |  |  | 1 |  |  |  |  |  |  |  |  |  |  |  |  |  |  |  | 3 |  |
| Megachile sp. 8 |  |  |  |  |  |  |  | 1 |  | 1 |  |  |  |  |  |  |  |  |  |  |  |  |
| Melipona beecheii Bennett, 1831 |  |  | 3 |  |  |  |  | 13 |  | 1 | 1 |  |  |  | 1 |  |  | 3 | 4 | 1 | 4 | 1 |
| Melipona solani Cockerell, 1912 |  | 1 | 1 |  |  |  |  |  |  |  |  |  |  |  |  |  |  |  | 1 |  | 1 |  |
| Melitoma marginella Cresson, 1872 |  | 1 | 1 |  |  |  |  |  |  |  |  |  |  |  | 1 | 1 |  |  |  |  |  |  |
| Mesocheira bicolor Fabricius, 1804 |  |  | 1 |  |  |  |  |  |  | 1 |  |  |  |  |  |  |  |  |  |  |  |  |
| Neocorynura discolor Smith 1879 |  | 1 |  |  | 6 | 1 |  |  |  | 1 |  |  | 1 | 1 | 1 | 1 |  |  | 1 | 2 |  |  |
| Neocorynura pubescens Friese, 1917 | 12 |  |  | 1 | 25 | 2 |  |  |  | 1 | 2 | 2 | 4 | 6 | 17 | 7 |  |  |  |  |  | 1 |
| Neocorynura sp. 1 |  |  |  |  | 6 |  |  |  |  |  | 3 | 3 |  |  |  |  |  |  |  |  |  |  |
| Neocorynura sp. 2 |  |  |  |  | 3 |  |  |  |  |  |  |  |  |  |  | 3 |  |  |  |  |  |  |
| Paratrigona guatemalensis Schwarz, 1938 | 5 | 29 | 2 |  | 31 |  |  | 1 | 1 |  |  | 4 | 6 | 8 | 9 |  | 1 | 6 | 8 | 1 | 6 | 20 |
| Partamona bilineata Say, 1837 |  | 2 |  | 5 | 13 | 6 | 3 | 3 | 2 |  |  |  | 3 | 2 | 4 |  |  | 2 | 3 | 6 | 14 |  |
| Partamona orizabaensis Strand, 1919 | 2 | 14 | 3 |  |  |  |  |  |  |  | 1 |  |  | 1 | 1 |  | 1 | 2 | 6 |  | 6 | 1 |
| Plebeia jatiformis Cockerell, 1912 | 1 | 1 | 3 | 1 | 26 | 4 |  | 1 |  | 1 | 1 |  | 1 | 1 | 2 | 9 |  | 1 | 2 | 7 | 9 | 3 |
| Pseudoaugochloropsis sp. 1 | 1 | 1 |  |  | 2 |  |  |  |  |  |  |  | 1 |  |  |  |  |  | 1 |  | 1 | 1 |
| Rhathymus atitlanicus Ayala, Hinojosa-Díaz, Armas-Quiñónez, 2019 |  | 1 | 1 |  |  |  |  |  | 1 |  |  |  |  | 1 | 1 | 1 |  |  |  |  |  |  |
| Scaptotrigona mexicana Guérin-Méneville, 1845 |  | 50 | 31 |  | 25 | 8 |  |  |  | 4 | 6 | 2 | 8 | 7 | 5 |  |  | 4 | 5 | 7 | 59 | 7 |
| Tetragonisca angustula Latreille, 1825 | 8 | 27 | 10 |  | 10 | 1 | 2 | 4 | 2 | 4 | 1 | 1 | 3 |  | 2 | 1 |  | 6 | 15 | 6 | 8 | 17 |
| Tetrapedia maura Cresson, 1878 | 30 | 9 | 2 |  |  | 7 |  |  |  | 2 | 4 | 2 | 9 |  | 2 | 1 | 2 | 1 | 7 |  | 4 | 14 |
| Tetraloniella sp. 1 |  |  | 1 |  | 1 |  |  |  |  |  |  |  |  |  |  |  | 1 | 1 |  |  |  |  |
| Thygater analis Lepeletier, 1841 | 1 |  |  |  |  |  |  |  |  |  |  |  |  |  |  |  | 1 |  |  |  |  |  |
| Trigona acapulconis Strand, 1919 |  | 1 | 1 |  |  |  |  |  |  |  |  |  |  |  |  |  |  |  |  | 1 | 1 |  |
| Trigona fulviventris Guérin-Méneille, 1845 | 61 | 83 | 19 | 13 | 42 | 1 | 13 | 1 | 6 | 8 | 15 | 12 | 10 | 9 | 38 | 12 | 35 | 12 | 34 | 16 | 17 | 21 |
| Trigona nigerrima Cresson, 1828 | 12 | 41 | 18 | 14 | 152 | 3 | 2 | 3 |  | 18 | 2 | 5 | 3 | 2 | 16 | 8 |  | 14 | 14 | 18 | 54 | 91 |
| Trigonisca pipioli Ayala, 1999 |  |  | 1 |  |  |  |  |  |  |  |  |  |  |  |  |  |  |  |  |  | 1 |  |
| Xylocopa fimbriata |  |  |  |  |  |  |  | 1 |  |  |  |  |  |  |  |  |  |  |  |  |  | 1 |
| Xylocopa guatemalensis Cockerell, 1912 |  |  |  |  |  |  |  |  | 1 |  | 1 |  |  |  |  |  |  |  |  |  |  |  |
| Xylocopa mexicanorum Cockerell, 1912 |  |  |  | 1 | 3 |  | 1 | 6 |  | 3 |  |  |  | 1 |  |  |  | 1 | 5 |  | 1 |  |
| Xylocopa muscaria Fabricius, 1775 |  |  |  |  | 2 |  | 1 | 4 |  | 1 | 1 | 2 |  |  |  |  |  | 2 | 1 |  |  |  |
| Xylocopa tabaniformis Smith, 1854 |  |  | 1 |  | 5 | 1 |  | 1 | 5 | 2 | 1 |  |  |  |  |  |  | 2 | 7 |  |  | 1 |
| Xylocopa viridis Smith, 1854 |  | 2 | 1 |  |  |  |  |  |  |  | 1 |  |  |  |  |  |  |  |  | 1 | 1 |  |
| Apidae no. 1 |  | 2 |  |  |  |  |  |  |  |  |  |  |  |  |  |  |  |  | 2 |  |  |  |
| Apidae no. 2 |  | 1 |  |  |  |  |  |  |  |  | 1 |  |  |  |  |  |  |  |  |  |  |  |
| Apidae no. 3 |  |  |  |  | 3 |  |  |  |  | 1 |  |  |  |  |  |  |  |  | 2 |  |  |  |
| Apidae no. 4 |  |  |  |  |  |  |  |  | 1 |  |  |  |  |  |  |  | 1 |  |  |  |  |  |
| Apidae no. 5 | 1 |  |  |  | 3 |  |  |  |  |  |  |  | 1 |  |  |  |  | 1 |  | 2 |  |  |
| Apidae no. 6 |  |  |  |  | 1 |  |  |  |  |  |  |  |  |  |  |  | 1 |  |  |  |  |  |
| **TOTAL NUMBER BEES** | 228 | 605 | 169 | 132 | 935 | 118 | 396 | 301 | 120 | 206 | 94 | 140 | 142 | 143 | 218 | 154 | 127 | 263 | 333 | 283 | 464 | 437 |
| **TOTAL NUMBER SPECIES** | 26 | 47 | 33 | 12 | 69 | 19 | 22 | 23 | 20 | 27 | 26 | 32 | 30 | 30 | 32 | 22 | 26 | 34 | 37 | 26 | 49 | 43 |
