# Supplementary material for: Bee diversity in secondary forests and coffee plantations in a transition between foothills and highlands in the Guatemalan Pacific Coast
Source: PeerJ. 2020 Jun 4;8:e9257. doi: 10.7717/peerj.9257 (PMC7276151; doi:10.7717/peerj.9257)
Supplement: Supplemental Information 3 [file peerj-08-9257-s003.docx]

**Appendix 3.** Plant species flowering during the study month and year.

| Plant species | 2013 | | | | | | | | | | 2014 | | |
| --- | --- | --- | --- | --- | --- | --- | --- | --- | --- | --- | --- | --- | --- |
|  | **3** | **4** | **5** | **6** | **7** | **8** | **9** | **10** | **11** | **12** | **1** | **2** | **3** |
| *Acmella oppositifolia* (Lamarck)  R.K. Jansen | | |  |  |  |  |  |  | X |  |  |  |  |
| *Aeschynomene hystrix* Poir. | | |  |  |  |  | X | X |  |  |  |  |  |
| *Ageratina* sp. 1 | |  |  |  |  |  |  |  |  |  | X |  |  |
| *Astragalus guatemalensis* Poir. | | |  |  |  |  |  | X |  |  |  |  |  |
| *Bidens* sp. 1 |  |  | X | X | X | X | X | X | X | X | X |  |  |
| *Bonplandia geminiflora* Cav. | | |  |  |  |  |  |  |  |  | X |  |  |
| *Borreria laevis* (Lam.) Griseb. | |  |  | X |  | X |  | X | X |  |  |  |  |
| *Boussingaultia racemosa* (Moq.)  Hemsl. | | |  |  |  |  |  |  |  |  |  | X | X |
| *Calea integrifolia* (DC). Hemsl. | |  |  |  |  |  |  |  |  | X |  |  |  |
| *Chromolaena* sp. 1 | |  |  |  |  |  |  |  |  | X |  |  |  |
| *Coffea arabica* L. | |  |  |  |  |  |  |  |  |  |  |  | X |
| *Commelina erecta* L. | |  |  |  | X | X | X | X | X | X |  |  |  |
| *Conyza* sp. 1 | | X |  |  |  |  |  | X |  |  |  |  |  |
| *Crotalaria vitellina* Ker.Gawl. | |  |  |  |  |  |  | X |  |  |  |  |  |
| *Crusea hispida* (Mill.) B.L. Rob. | |  | X | X | X | X |  | X |  |  |  |  |  |
| *Crusea setosa* (M. Martens &  Galeotti) Standl. & Steyerm. | | X | X | X | X | X |  | X |  |  |  |  |  |
| *Desmodium nicaragüense* Oerst. | | |  |  |  |  |  |  | X |  |  |  |  |
| *Desmodium scorpiurus* (Sw.)  Desv. | | |  |  |  |  |  | X | X | X |  |  |  |
| *Dahlia australis* (Sherff) P. D.  Sørensen. | |  |  |  |  |  |  |  | X | X | X | X | X |
| *Dicliptera inutilis* Leonard. | |  |  |  |  |  |  |  |  |  |  | X |  |
| *Elephantopus mollis* Kunth. | | |  |  |  |  | X | X |  | X |  |  |  |
| *Eupatorium* sp. 1 | |  |  |  |  |  |  |  |  |  | X | X | X |
| *Eupatorium* sp. 2 | X | X |  |  |  |  |  |  |  |  |  |  | X |
| *Euphorbia graminea* Jacq. | | |  |  |  |  |  |  |  | X |  |  |  |
| *Galinsoga quadriradiata* Ruiz & Pav | | |  |  | X |  | X |  |  | X | X |  |  |
| *Gnaphalium liebmannii* Sch. Bip. | | X |  |  |  |  |  |  |  |  |  |  |  |
| *Heterocentron subtriplinervium*  (Link & Otto) A. Braun & C. D.  Bouché | | | |  |  |  |  |  |  | X |  |  |  |
| *Hieracium* sp. 1 | |  | X |  |  |  |  |  |  |  |  |  |  |
| *Hyptis urticoides* Kunth. | |  |  |  |  |  |  |  | X | X |  |  |  |
| *Inga micheliana* Harms. | |  |  |  |  |  |  |  |  |  |  | X |  |
| *Ipomea tiliacea* (Willd.) Choisy | X | X |  |  |  |  |  |  |  |  |  |  |  |
| *Ipomoea aristolochiaefolia* G Don | X |  |  |  |  |  | X |  |  |  |  |  |  |
| *Ipomoea cholulensis* (Kunth) G. Don | | |  |  |  |  |  |  |  | X |  |  |  |
| *Ipomoea dumosa* (Benth.)  L. O. Williams | |  |  |  |  |  |  |  | X | X |  |  |  |
| *Ipomoea purga* (Wender.) Hayne | |  |  |  |  | X |  |  |  |  | X |  |  |
| *Ipomoea silvicola* House | |  | X | X | X | X | X | X | X |  |  |  |  |
| *Ipomoea squamosa* Choisy | |  |  |  |  |  |  |  | X | X |  | X | X |
| *Ipomoea tiliácea* (Wild.)Choisy | |  |  |  |  |  |  |  |  |  | X |  |  |
| *Iresine grandis* Standl. | |  |  |  |  |  |  |  |  |  | X |  |  |
| *Justicia aurea* Schltdl. | X |  |  |  |  |  |  |  |  |  |  | X | X |
| *Lippia myriocephala* Schltdl. &  Cham. | |  |  |  |  |  |  |  |  |  |  |  |  |
| *Lippia cardiostegia* Benth. | |  |  |  |  |  |  |  | X | X |  |  |  |
| *Lobelia laxiflora* Kunth. | X | X | X |  |  |  |  |  |  | X | X | X | X |
| *Melampodium paniculatum*  Gardner. | | | | X | X | X | X |  |  |  |  |  |  |
| *Melampodium* sp. 2 | | |  |  |  |  | X |  |  |  |  |  |  |
| *Melanthera nívea* (L.) Small | |  |  |  |  |  |  |  |  | X | X |  |  |
| *Mimosa albida* Humb. & Bompl.  Ex Willd. | |  |  |  | X | X | X | X | X |  |  |  |  |
| *Montanoa guatemalensis* B. L. Rob.  & Greenm. | | |  |  |  |  |  |  |  | X | X |  |  |
| Asteraceae 1 | |  |  |  |  |  |  | X |  |  |  |  |  |
| Asteraceae 6 | |  |  |  |  |  |  |  |  |  | X |  |  |
| Asteraceae 7 | |  |  |  |  |  |  |  | X |  |  |  |  |
| Asteraceae 8 | |  |  |  |  |  |  |  | X |  |  |  |  |
| *Oenothera rosea* Aiton | X |  |  | X |  |  |  |  |  |  |  |  |  |
| *Oxalis corniculata* L. | |  |  | X | X |  |  |  |  |  |  |  |  |
| *Oxyrhynchus trinervius* L. | | |  |  |  |  |  |  | X |  |  |  |  |
| *Perymenium ghiesbreghtii* B. L.  Rob. & Greenm. | | |  |  |  |  |  | X |  |  |  |  |  |
| *Perymenium grande* Hemsl. | X | X | X |  |  |  |  |  |  |  |  |  | X |
| *Phytolacca icosandra* L. | | X |  |  |  |  |  |  |  |  |  |  |  |
| *Phytolacca rivinoides* Kunth &  Bouse | | |  |  |  |  |  |  |  | X |  | X | X |
| *Podachaenium eminens* (Lag.) Sch.Bip | X | X | X |  |  |  |  |  |  |  |  | X | X |
| *Pseudelephantopus spicatus*  (Juss. ex Aubl.) C.F. Baker | | | |  |  |  |  |  |  | X | X |  |  |
| *Quamoclit cholulensis* (Kunth) G. Don | X |  |  |  |  |  |  |  |  |  |  |  |  |
| *Quamoclit hederifolia* (L.) G. Don | | |  |  |  |  |  |  |  | X | X |  |  |
| *Ricinus communis* L. | X |  |  |  | X | X |  | X | X | X | X | X | X |
| *Roldana petasioides* Greenm. ex Donn. Sm. | X |  |  |  |  |  |  |  |  |  |  |  |  |
| *Ruellia donnell-smithii* Leonard | X |  |  |  |  |  |  |  |  |  |  |  | X |
| *Salvia misella* Kunth | |  |  |  |  |  |  |  |  |  |  |  | X |
| *Salvia mocinoi* Benth. | |  |  |  |  |  |  |  |  | X | X | X |  |
| *Salvia occidentalis* Sw. | |  |  |  |  |  |  |  |  | X |  | X | X |
| *Salvia polystachia* Cav. | |  |  |  |  |  |  |  | X | X |  |  |  |
| *Schistocarpha* sp. 1 | X |  |  |  |  |  |  |  |  | X | X |  | X |
| *Schistocarpha* sp. 2 | |  |  |  |  |  |  |  |  | X | X |  |  |
| *Schistocarpha* sp. 3 | |  |  |  |  |  |  |  |  | X | X | X |  |
| *Senecio chenopodioides* Kunth. | | |  |  |  |  |  |  |  |  | X |  |  |
| *Senecio* sp. 1 | |  |  |  |  |  |  |  |  |  |  | X |  |
| *Sida rhombifolia* L. | X | X | X | X |  |  | X | X | X | X | X |  |  |
| *Solanum americanum* Mill. | X | X | X | X |  |  |  | X | X | X | X | X | X |
| *Solanum hartwegii* Benth. | X | X | X |  |  |  |  |  |  |  | X | X | X |
| *Sonchus oleraceus* L. | | X | X | X | X | X | X |  |  |  | X |  |  |
| *Spilanthes* sp. 2 | |  | X | X | X | X | X | X |  |  |  |  |  |
| *Stylosanthes* sp.1 | |  |  |  |  |  |  | X |  |  |  |  |  |
| *Tagetes tenuifolia* Cav. | |  |  |  |  |  |  | X |  |  |  |  |  |
| *Tecunumania quetzalteca* Standl.  & Steyerm. | | X | X |  |  |  |  |  |  |  |  |  |  |
| *Tephrosia nicaraguensis* Oerst. | X | X | X |  | X | X | X | X | X | X | X | X |  |
| *Tinantia erecta* | |  |  |  |  |  |  | X |  |  |  |  |  |
| *Tithonia longiradiata* (Bertol.)  S.F.Blake | | |  |  |  |  |  | X | X | X |  |  |  |
| Tradescantia aff. crassifolia | | | |  |  |  |  | X |  |  |  |  |  |
| *Tridax procumbens L.* | | X |  | X |  | X |  |  |  |  |  |  |  |
| *Tripogandra amplexicaulis*  (Klotzsch ex C.B. Clarke) Woodson | | |  |  |  |  | X |  |  |  |  |  |  |
| *Trixis inula* Crantz. | X | X |  |  |  |  |  |  |  |  |  | X | X |
| *Urochloa humidicola* (Rendle)  Morrone & Zuloaga | | |  |  |  | X | X |  |  |  |  |  |  |
| *Valeriana* sp. 1 | |  |  |  |  |  |  | X |  |  |  |  |  |
| *Verbena carolina* L. | | X | X | X | X | X | X | X |  | X | X |  | X |
| *Verbesina scabriuscula* S.F. Blake | | |  |  |  |  |  | X |  |  |  |  |  |
| *Verbesina* sp. 1 | |  |  |  |  |  |  |  |  | X |  |  |  |
| *Vernonia deppeana* Less. | |  |  |  |  |  |  |  |  |  |  | X |  |
| *Wigandia urens* (Ruiz & Pav.) Kunth | X | X | X | X |  |  |  |  | X | X | X | X | X |
| *Witheringia affinis* (C.V. Morton)  Hunz. | |  |  |  |  |  |  |  |  |  |  | X | X |
| *Youngia japónica* (L.) DC. | |  |  |  |  |  |  |  |  | X |  |  |  |
